# The Effects of Combined Laser Cladding and Layer-by-Layer Remelting on the Forming Quality and Properties of H13 Steel Clad Layers

**DOI:** 10.3390/ma19194196

**Published:** 2026-09-30

**Authors:** Yifeng Zhao, Rui Wang

**Affiliations:** School of Mechanical Engineering, Shijiazhuang Tiedao University, Shijiazhuang 050043, China; zhaoyifeng2024@163.com

**Keywords:** H13 steel, laser cladding, layer-by-layer remelting, microstructure, mechanical properties

## Abstract

To improve the forming quality, microstructural characteristics, and mechanical properties of multi-track multilayer laser-clad H13 steel structures, a combined laser cladding and layer-by-layer remelting process was employed. The basic cladding parameters were comprehensively evaluated using an orthogonal experimental design combined with the entropy weight method–gray relational analysis (EWM-GRA), while the remelting parameters were determined through a full-factorial experiment. Multi-track multilayer specimens with and without layer-by-layer remelting were subsequently compared in terms of forming characteristics, microstructure, and mechanical properties. Within the investigated parameter range, the preferred cladding parameter combination was a laser power of 1300 W, scanning speed of 12 mm/s, and powder feed rate of 13 g/min, while the preferred remelting parameter combination was 780 W and 18 mm/s. Layer-by-layer remelting produced a smoother surface with less pronounced inter-track overlap traces. Qualitative examination of the cross-sections suggested a tendency toward fewer locally observed pores and reduced variation in microstructural morphology among regions at different heights. The average microhardness increased from 756.9 HV to 789.5 HV, while the ultimate tensile strength and yield strength increased from 1524.23 MPa and 1173.37 MPa to 1604.46 MPa and 1342.88 MPa, respectively. The increased strength was accompanied by reduced elongation after fracture, from 2.57% to 1.41%. These results provide a reference for process parameter selection and property tailoring of layer-by-layer remelting in multi-track multilayer laser-clad H13 steel structures.

## 1. Introduction

H13 steel (4Cr5MoSiV1) is a widely used hot-work die steel that combines good high-temperature strength, hot hardness, and thermal fatigue resistance, making it suitable for critical components of hot-work dies, such as die-casting and hot-forging dies [1,2,3]. During long-term service, the die surface is subjected to high temperatures and complex thermo-mechanical cyclic loading, which may lead to wear, thermal cracking, and local plastic deformation, resulting in progressive degradation of service performance and a shortened service life [4,5,6]. Therefore, the use of efficient and reliable surface strengthening and remanufacturing technologies [7,8] to restore damaged regions and improve the service reliability of H13 steel [9,10] is important for extending the service life of hot-work dies.

Laser cladding is an important surface modification and remanufacturing technique [11,12], in which a high-energy laser beam is used to simultaneously melt the alloy powder and the surface layer of the substrate [13], followed by rapid solidification to form a clad layer metallurgically bonded to the substrate [14]. Compared with conventional surfacing and thermal spraying processes, laser cladding offers several advantages, including a small heat-affected zone [15], relatively low dilution, and strong metallurgical bonding [16]. It has therefore been widely applied to the repair and performance enhancement of die steels, aerospace components [17,18], and energy equipment [19]. However, owing to the rapid melting–solidification process and complex thermal cycles [20,21], laser-cladded layers may still exhibit surface undulations, pores [22], residual stresses [23], and microstructural inhomogeneity [24]. This issue is particularly relevant for multi-track multilayer structures, where interlayer heat accumulation [25] and repeated thermal cycling [26] result in different thermal histories in different regions, leading to variations in microstructure and properties along the deposition direction and consequently affecting forming quality, microstructural uniformity, and mechanical performance [27].

Laser remelting has been incorporated into laser cladding to further improve the forming quality and properties of clad layers [28]. In this process, the deposited layer is rescanned without powder feeding [29], inducing remelting and resolidification that modify molten pool flow, elemental distribution, and microstructural evolution [30,31]. Conventional post-process remelting is performed after deposition is complete, whereas in situ remelting is integrated into the deposition process. Layer-by-layer remelting is a specific form of in situ remelting in which each completed layer is remelted after deposition and before subsequent layer deposition, incorporating remelting throughout the build process.

Previous studies have investigated the effects of remelting on forming quality, microstructure, and material properties. Li et al. [32] found that an appropriate increase in remelting power could reduce the porosity and promote a more uniform elemental distribution in in situ WC-reinforced Ni-based coatings, whereas the effectiveness was limited under insufficient energy input. Zhao et al. [33] reported that the redistribution and spreading of molten materials driven by surface tension facilitated the reduction in surface roughness and improved the near-surface densification of Ni-based cladding layers. Wu et al. [34] further emphasized that the appropriate matching of remelting power and scanning speed is a critical factor in improving the forming quality of Stellite 6/WC cladding layers.

Remelting can also modify microstructure and material properties. Singh et al. [35] refined the grains and reduced their aspect ratio in Inconel 718 deposits through layer-by-layer in situ remelting. Jiang et al. [36] reported a more uniform microstructure and fine secondary-phase precipitation in remelted Stellite 6 clad layers, accompanied by improvements in microhardness and wear resistance. Kaierle et al. [37] incorporated remelting into the repair of single-crystal blades by laser cladding, improving microstructural continuity through control of local solidification behavior. However, microstructural improvements do not necessarily translate into simultaneous increases in strength and ductility. Hodgir et al. [38] found that in situ remelting increased the yield strength and ultimate tensile strength of SS316L deposits while reducing elongation after fracture, highlighting the need to consider both strength and ductility when evaluating the effects of remelting. For H13 steel, various forms of laser remelting have also been investigated. Karmakar et al. [39] studied the effects of processing parameters on melt pool thermal history, microstructure, and hardness during laser surface remelting of H13 steel. Jung et al. [40] applied repeated laser scanning during H13 powder bed fusion to improve material densification and hardness. Bidare et al. [41] investigated interlayer laser remelting during H13 directed energy deposition and evaluated its effects on pores, hardness, and component deformation. These studies indicate that laser remelting has been applied in both surface treatment and additive manufacturing processes of H13 steel and can influence densification, microstructure, and hardness.

Although previous studies have applied laser remelting to H13 steel surface treatment, powder bed additive manufacturing, and directed energy deposition processes, existing works have mainly focused on substrate surface remelting, repeated laser scanning in powder bed fusion, or interlayer remelting conducted after several deposited layers. These findings do not fully clarify the effects of layer-by-layer remelting on multi-track multilayer H13 steel structures. However, for powder-fed multi-track multilayer H13 laser cladding structures, studies involving full-layer remelting without powder feeding after each deposited layer and further evaluating surface formation, local pore characteristics, microstructural morphology at different heights, microhardness, and tensile properties remain limited. During multi-track multilayer cladding, inter-track overlap and layer stacking jointly influence local thermal histories. Regions at different heights experience different numbers of reheating cycles and different heat dissipation conditions and may therefore respond differently to additional remelting. Therefore, existing findings cannot directly clarify the role of layer-by-layer full-layer remelting in such structures, and further investigation is required to evaluate its effects on microstructural variation along the build direction and overall mechanical properties. To address these issues, this study combines the optimization of single-track cladding and remelting parameters with the evaluation of multi-track multilayer H13 structures. Specimens produced with and without layer-by-layer remelting are compared in terms of surface formation, local pores, microstructural morphology at different heights, and changes in strength and ductility, The focus of this study is to evaluate the applicability of layer-by-layer full-layer remelting in multi-track multilayer H13 structures and its effects on microstructural and mechanical responses, rather than proposing laser remelting as a new processing method.

To achieve these objectives, this study employs a combined laser cladding and layer-by-layer remelting process, following a sequence of basic cladding parameter optimization, remelting parameter optimization, and validation using multi-track multilayer structures. First, an L16 (4^3^) orthogonal experimental design is used to select the basic cladding parameters. A two-factor, four-level full-factorial experiment is then conducted under the selected cladding conditions to determine the remelting parameters. The entropy weight method–gray relational analysis (EWM-GRA) is applied at both stages to jointly evaluate geometric forming characteristics, dilution ratio, microhardness, and surface roughness [42]. Weights are determined from the dispersion of each indicator, and parameter combinations are evaluated using the overall gray relational grade, allowing parameter selection to account for multiple responses rather than a single indicator. The selected parameters are subsequently used to fabricate multi-track multilayer specimens with and without layer-by-layer remelting. For the remelted specimens, each completed layer is allowed to cool and then remelted once over its entire surface along the original cladding path without powder feeding, before deposition of the next layer. Macroscopic morphology, microstructure at different heights, microhardness, tensile properties, and fracture morphology are compared to evaluate the effects of layer-by-layer remelting on the deposition quality, microstructure, and mechanical properties of multi-track multilayer H13 steel structures.

## 2. Materials and Methods

### 2.1. Materials and Experimental Equipment

H13 steel was used as the substrate material, while H13 steel powder was used as the cladding material. The substrate dimensions were 100 mm × 100 mm × 10 mm. Since no independent compositional analysis was performed for the substrate and the actual batch composition was unavailable, only the nominal chemical composition range of H13 steel is provided. No additional heat treatment was applied to the substrate before laser cladding. Prior to cladding, approximately a 100 mm × 80 mm area of the substrate surface was mechanically ground using a 120-grit alumina abrasive wheel to remove the oxide layer and surface contaminants until a metallic luster was obtained, followed by cleaning with acetone to remove residual oil and impurities. To minimize the effects of moisture absorption and particle agglomeration on powder-feeding stability and cladding quality, the H13 steel powder was dried in a vacuum oven at 120 °C for 2 h before the experiments. The particle size of the cladding powder ranged from 51 to 85 μm, as measured from SEM images of the powder used in this study. The nominal chemical composition range of H13 steel is provided in Table 1.

**Figure 1:**
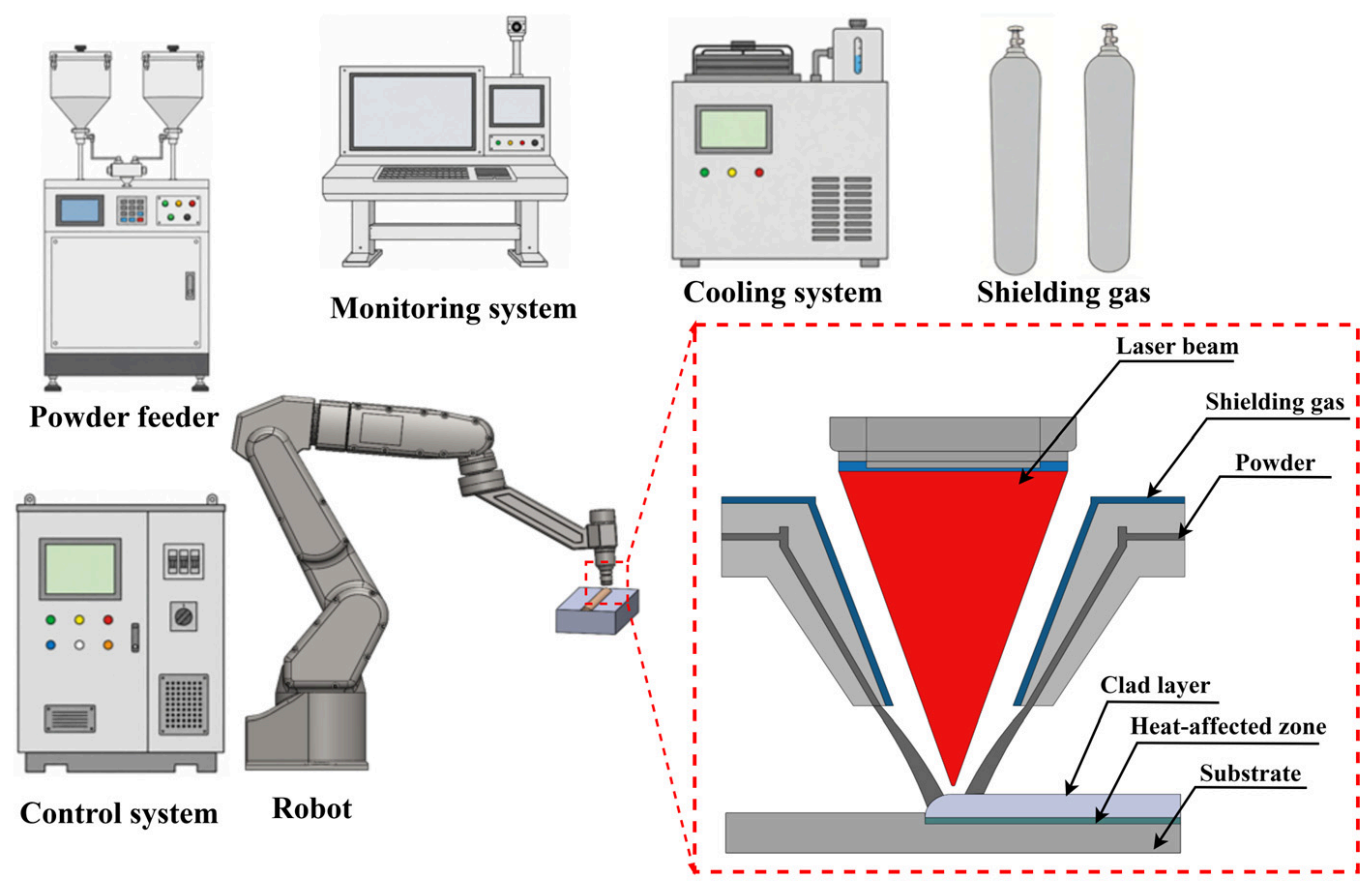
Schematic diagram of laser cladding system.

The laser cladding experiments were conducted using a coaxial powder-feeding laser cladding system, as schematically illustrated in Figure 1. The system mainly consisted of a Keplin CWX6000 continuous-wave fiber laser (Beijing BWT Photonics Technology Co., Ltd., Beijing, China), a laser head, a powder-feeding system, an industrial robot, and a cooling system. During cladding, the laser head height was 16.5 mm and the laser spot diameter was 4 mm. Argon with a purity of 99% was used as both the shielding gas and the powder carrier gas, with flow rates of 15 L/min and 10 L/min, respectively.

### 2.2. Experimental Methods

**Figure 2:**
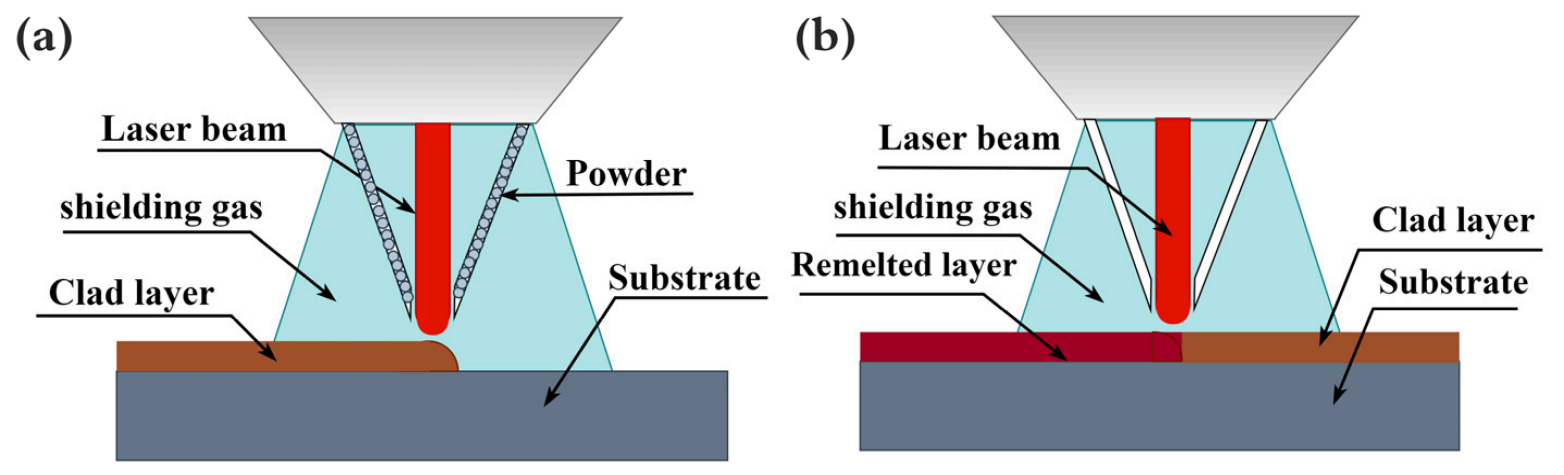
Schematic illustration of laser cladding and remelting processes: (**a**) laser cladding; (**b**) laser remelting.

The experiments were conducted following the sequence of basic cladding parameter optimization–remelting parameter optimization–multi-track multilayer validation. First, single-track laser cladding experiments were performed to optimize the basic cladding parameters. Subsequently, single-track remelting experiments were conducted using the optimized cladding parameters to determine suitable remelting parameters for the subsequent layer-by-layer remelting process. Finally, the optimized cladding and remelting parameters were used to fabricate multi-track multilayer specimens, and the forming quality, microstructural characteristics, and mechanical properties of the specimens produced with and without layer-by-layer remelting were comparatively investigated. The laser cladding and remelting processes are illustrated in Figure 2.

#### 2.2.1. Orthogonal Experimental Design

To obtain basic cladding parameters suitable for subsequent remelting and the fabrication of multi-track multilayer specimens, an L16 (4^3^) orthogonal experimental design was adopted, with laser power, scanning speed, and powder feed rate selected as the main factors, each at four levels. The laser powers were set to 1000, 1300, 1600, and 1900 W; the scanning speeds were 10, 12, 14, and 16 mm/s; and the powder feed rates were 9, 11, 13, and 15 g/min. A total of 16 single-track cladding experiments were designed. The factors and their parameter combinations are listed in Table 2.

#### 2.2.2. Remelting Experimental Design

Based on the selected basic cladding parameters obtained from the orthogonal and confirmation experiments, namely a laser power of 1300 W, a scanning speed of 12 mm/s, and a powder feed rate of 13 g/min, single-track laser remelting experiments were subsequently conducted. The remelting laser power *Pr* and scanning speed *Vr* were selected as the main variables. The remelting laser power was set at 780, 910, 1040, and 1170 W, while the remelting scanning speed was set at 14, 16, 18, and 20 mm/s. A two-factor, four-level full-factorial experimental design was employed, resulting in a total of 16 parameter combinations, as listed in Table 3.

#### 2.2.3. Fabrication of Multi-Track Multilayer Specimens and Layer-by-Layer Remelting

Based on the optimized cladding and remelting parameters, multi-track multilayer specimens were fabricated with and without layer-by-layer remelting. The deposition area of both groups was 60 mm × 40 mm, with an inter-track overlap ratio of 40%. All tracks were deposited using unidirectional parallel scanning, with a total of four layers. For the specimens without remelting, deposition was completed layer by layer according to the prescribed cladding path without any additional remelting treatment. For the layer-by-layer remelted specimens, after each deposited layer was completed, the surface temperature of the deposited layer was measured using a VICTEKNIX V300 infrared thermometer (Shenzhen VICTEKNIX Technology Co., Ltd., Shenzhen, China). The layer was naturally cooled to 200 °C and then remelted once along the original cladding path under powder-feeding-off conditions, with the remelting direction kept consistent with the cladding direction. The remelting tracks followed the same overlap strategy as the original cladding tracks, with an inter-track overlap ratio of 40%. Subsequently, the effects of layer-by-layer remelting on the multi-track multilayer structures were comparatively evaluated in terms of macroscopic morphology, microstructure, microhardness, and tensile properties.

### 2.3. Comprehensive Evaluation Method

To comprehensively evaluate the multiple response indicators of the clad layers under different processing conditions, the entropy weight method–gray relational analysis (EWM-GRA) was employed [42]. The entropy weight method determines the objective weight of each evaluation indicator based on the degree of dispersion of the corresponding data, while gray relational analysis further calculates the degree of association between each experimental scheme and the ideal reference sequence to obtain the gray relational grade (GRG), which represents the comprehensive performance of each scheme. Clad height H, clad width W, dilution ratio η, microhardness HV, and surface roughness Sa were selected as the evaluation indicators. Among them, H and Sa were treated as smaller-the-better indicators, whereas W and HV were treated as larger-the-better indicators. Within the investigated parameter range, a smaller clad height is beneficial for limiting excessive reinforcement height while satisfying the requirements of subsequent inter-track overlapping and multilayer deposition. Considering that the substrate and powder are both H13 steel, an appropriate dilution ratio is beneficial for ensuring metallurgical bonding between the clad layer and substrate, whereas excessive dilution may enhance the influence of substrate material on the composition and properties of the clad layer. Based on previous studies involving the same H13 substrate/H13 powder system [43], a dilution ratio of 30% was adopted as the reference target value in this study rather than an absolute optimum value universally applicable to H13 laser cladding systems, and the deviation was defined as follows:

(1) $$D_{\eta_{i}}=\left|\eta_{i}-\eta_{0}\right|,\eta_{0}=30\%$$

where *η*_i_ is the dilution ratio value of the *i*th specimen and *η*_0_ is the target dilution ratio. The dilution deviation *D_η_* was treated as a smaller-the-better indicator. To eliminate the effects of different dimensions and numerical ranges among the evaluation indicators, the original data were first normalized according to the optimization direction of each indicator. Subsequently, entropy weights were calculated based on the dispersion degree of the normalized data, and gray relational analysis was performed to determine the association degree between each experimental scheme and the ideal reference sequence. The distinguishing coefficient in the gray relational analysis was set to 0.5. The basic cladding and remelting datasets were normalized and entropy-weighted, and subjected to gray relational analysis independently. The GRG was used as the overall evaluation index, with a higher GRG indicating that the corresponding parameter combination is closer to the ideal reference state.

### 2.4. Characterization and Mechanical Testing

#### 2.4.1. Measurement of Geometrical Dimensions and Dilution Ratio

**Figure 3:**
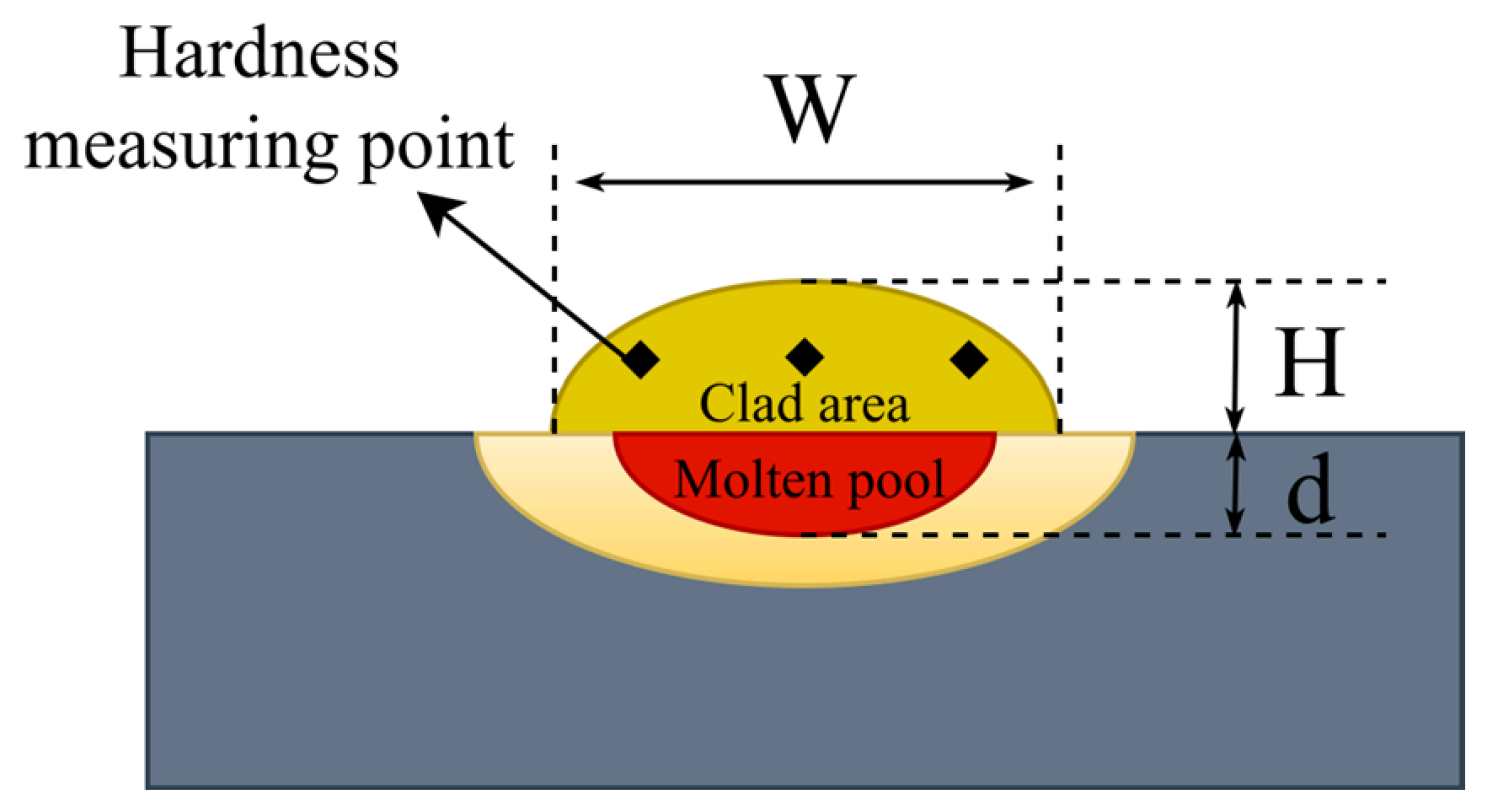
Schematic illustration of cross-section of clad layer.

One cross-section was extracted from the stable forming region of each clad track. After metallographic preparation, the cross-sectional optical images were obtained using a ZEISS Axio Observer.A1m metallographic microscope (Carl Zeiss Microscopy GmbH, Jena, Germany). Low-magnification optical micrographs of single-track clad layers were acquired at 20× magnification. The clad height H, clad width W, and penetration depth d were measured from cross-sectional metallographic images using ImageJ software (version 1.54f). Each geometrical parameter was measured three times on the same cross-sectional image, and the average value was taken as the measurement result for each specimen. The definitions of the geometrical parameters are shown in Figure 3. Considering the irregular cross-sectional shape of the clad layer, the dilution ratio η was calculated using the geometric height method as follows:

(2) $$\eta (\%)=\frac{d}{H+d}\times 100\%$$

#### 2.4.2. Microstructural Characterization

The microstructures of different regions of the clad layers were observed using a ZEISS Axio Observer.A1m metallographic microscope (Carl Zeiss Microscopy GmbH, Jena, Germany). Cross-sectional specimens with dimensions of approximately 10 mm × 10 mm × 10 mm were cut from the stable forming region of the cladded specimens using wire electrical discharge machining. The specimens were sequentially ground with 200# to 2000# SiC abrasive papers and then mechanically polished using a 1 μm diamond suspension. After polishing, the specimens were etched using a 4 vol.% nital solution for approximately 15 s, followed immediately by cleaning with absolute ethanol and drying.

#### 2.4.3. Mechanical Property Testing

Vickers microhardness testing: Microhardness measurements were performed using a Naibo THVS-M-AXYZF microhardness tester (Jiashan Naibo Precision Instrument Co., Ltd., Jiaxing, China) under a load of 500 gf and a dwell time of 10 s. For the single-track clad layers, three measurement points were selected on the cross-section of each clad layer, as shown in Figure 3, and the mean value was used as the microhardness of the corresponding specimen. For the multi-track multilayer clad layers, five measurement points were arranged at intervals of 0.5 mm from the top of the clad layer toward the substrate to characterize the microhardness distribution along the thickness direction. The average of the five measurements was taken as the mean microhardness of each specimen. The average value of the five measurements was only used to represent the overall hardness level of the measured region. The individual measurement points were used to characterize the spatial hardness variation within the same specimen and were not considered as independent specimen replicates.

**Figure 4:**
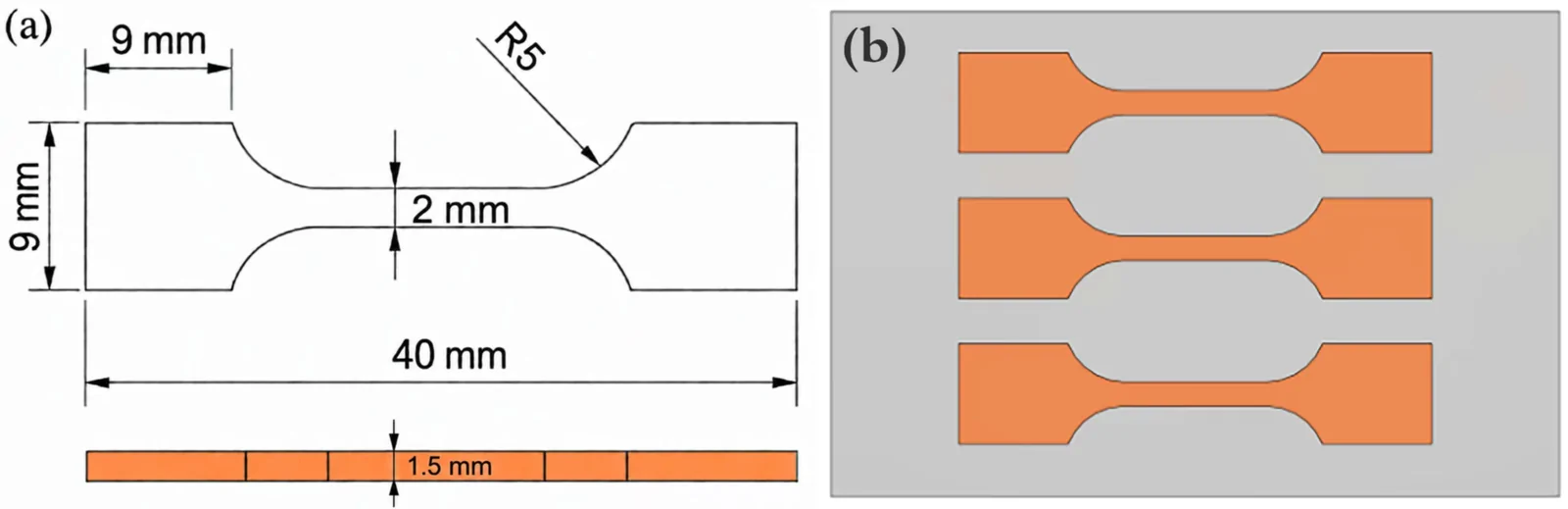
Tensile specimen preparation scheme: (**a**) dimensions of tensile specimen (mm); (**b**) sampling layout.

Tensile testing: Tensile specimens with a thickness of 1.5 mm were machined from the multi-track multilayer specimens with and without layer-by-layer remelting using wire electrical discharge machining. The specimen dimensions and sampling locations are shown in Figure 4. Tensile tests were conducted at room temperature using an Instron 3369 universal testing machine (Instron, Norwood, MA, USA) at a loading rate of 1 mm·min^−1^ until fracture. Three replicate specimens were tested for each processing condition, and the ultimate tensile strength, yield strength, and elongation after fracture were reported as the mean values with standard deviations calculated from the three measurements.

#### 2.4.4. Surface Morphology and Roughness Measurement

The three-dimensional surface morphology of the clad layers was characterized using a BRUKER npflex 3D white-light interferometer (Bruker Corporation Headquarters, Billerica, MA, USA), and the areal arithmetic mean height Sa was used to evaluate the surface roughness. Considering the non-uniformity of the clad surface morphology, measurements were performed at three fixed locations along the length direction within the stable forming region of each clad track, namely, the left, middle, and right regions. The actual measurement area at each location was approximately 1.7 mm × 1.4 mm. Before calculating Sa, the three-dimensional height data were subjected to plane correction to eliminate the overall slope caused by specimen mounting inclination. No additional form removal or spatial filtering was applied except for plane correction. The average Sa value from the three locations was taken as the final surface roughness result.

## 3. Results and Discussion

### 3.1. Orthogonal Experimental Results and Optimization of Basic Cladding Parameters

**Figure 5:**
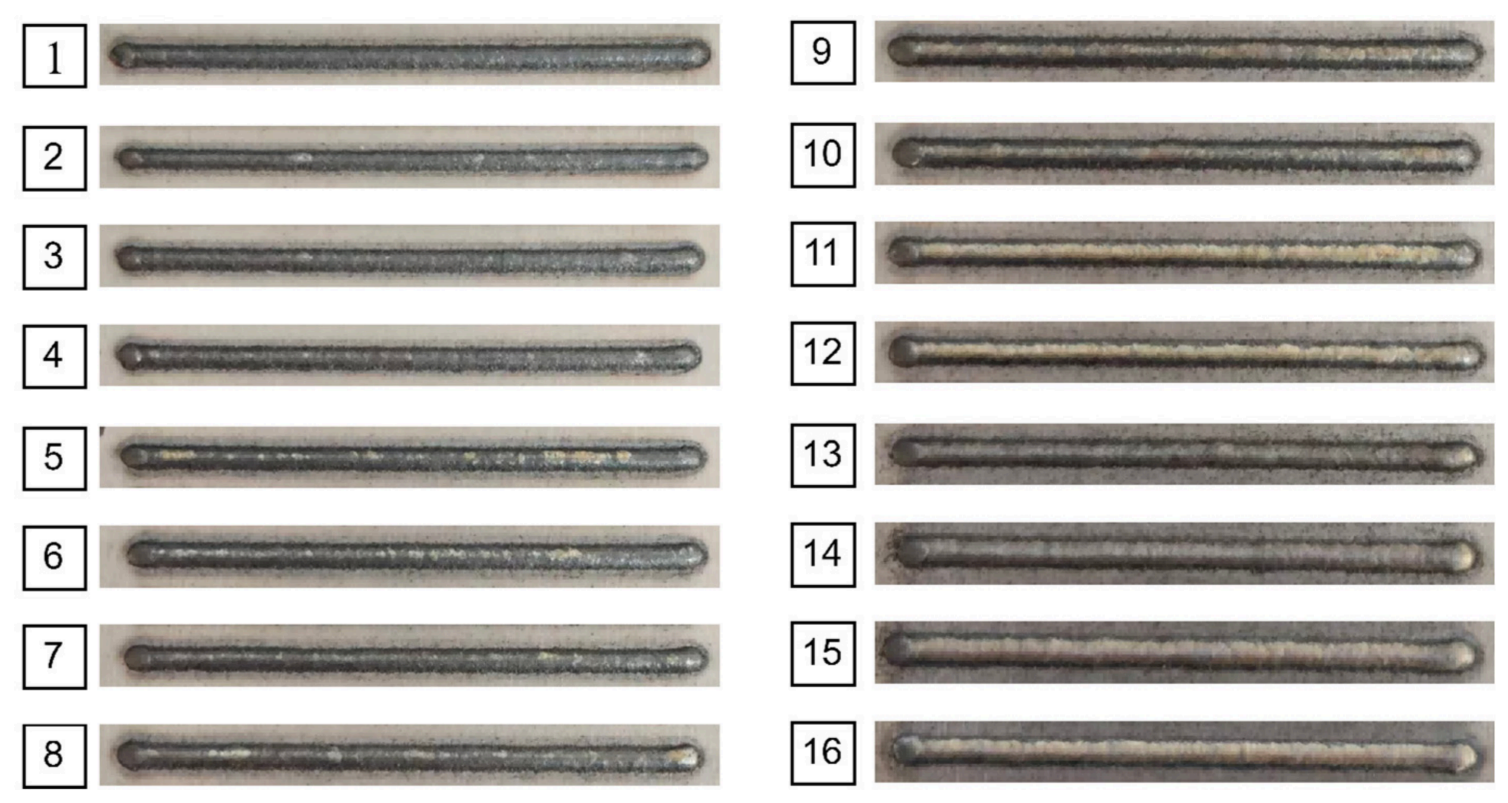
Macroscopic surface morphologies of 16 single-track cladding specimens.

Figure 5 shows the macroscopic morphologies of the 16 single-track cladding specimens. Continuous clad tracks were obtained for all parameter combinations, although differences were observed in surface flatness, track uniformity, and edge formation. Ripple-like surface undulations and adhered powder particles were visible on some specimens, indicating that different processing parameter combinations exerted a noticeable influence on track formation and molten pool stability.

Table 4 presents the clad height H, clad width W, microhardness HV, surface roughness Sa, and dilution ratio η for each specimen (see Table S1 for full experimental data). The response indicators varied noticeably among the different parameter combinations, making it difficult to determine the basic cladding parameters based on a single indicator alone. Therefore, the EWM-GRA method was employed for comprehensive evaluation.

**Figure 6:**
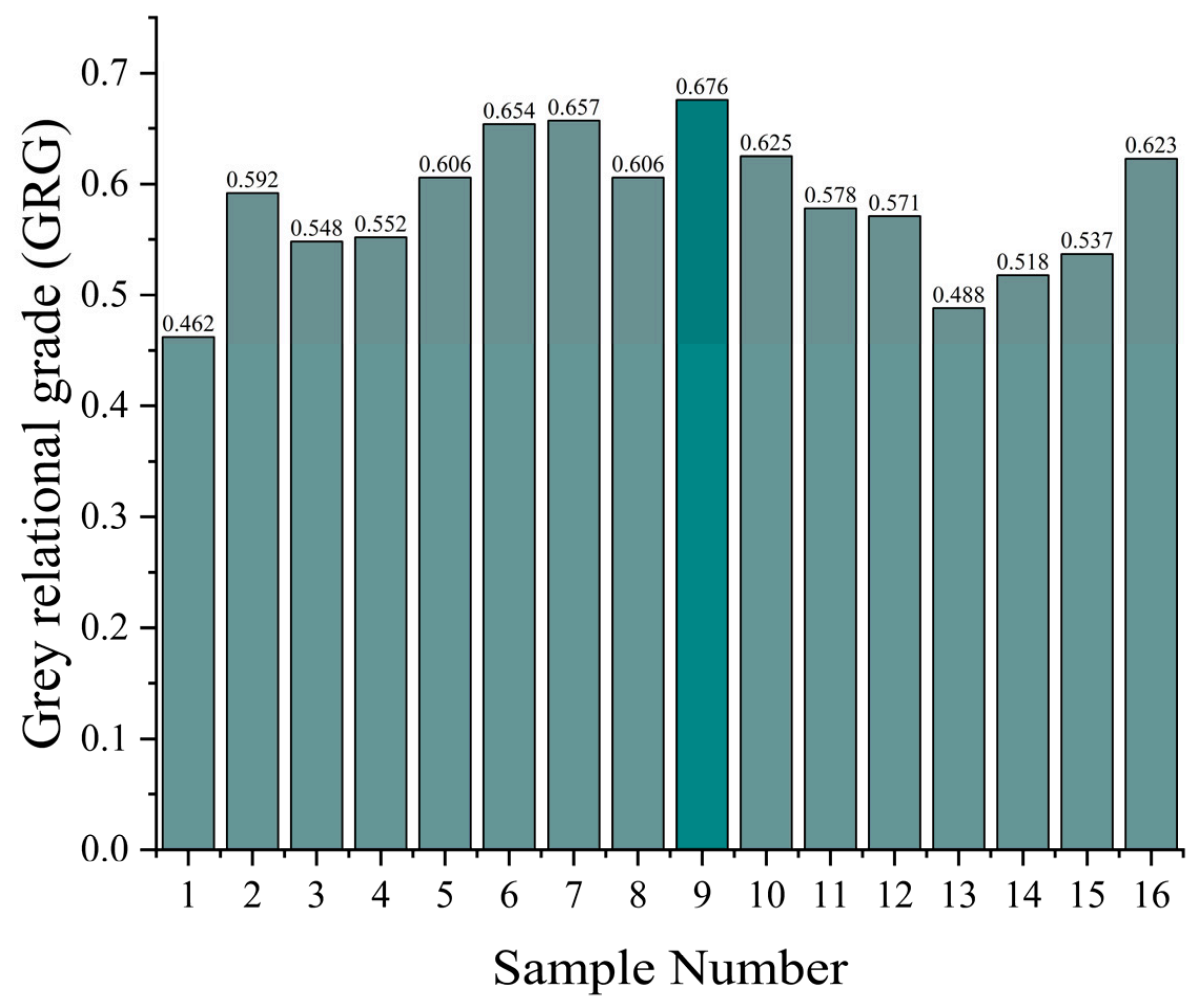
The distribution of the gray relational grade (GRG) for the 16 specimens.

The gray relational grades (GRGs) of the 16 specimens are shown in Figure 6. Considerable differences in GRGs were observed among the specimens, with specimen No. 9 exhibiting the highest value of 0.676. This indicates that this parameter combination achieved a relatively favorable balance among clad geometry, microhardness, surface roughness, and dilution ratio. However, because the factor levels in the orthogonal design were combined according to a predefined experimental matrix, the parameter combination corresponding to the highest GRG in a single experiment does not necessarily represent the optimal combination of factor levels based on the main effects. Therefore, a main-effect analysis was further conducted using GRG as the response.

**Figure 7:**
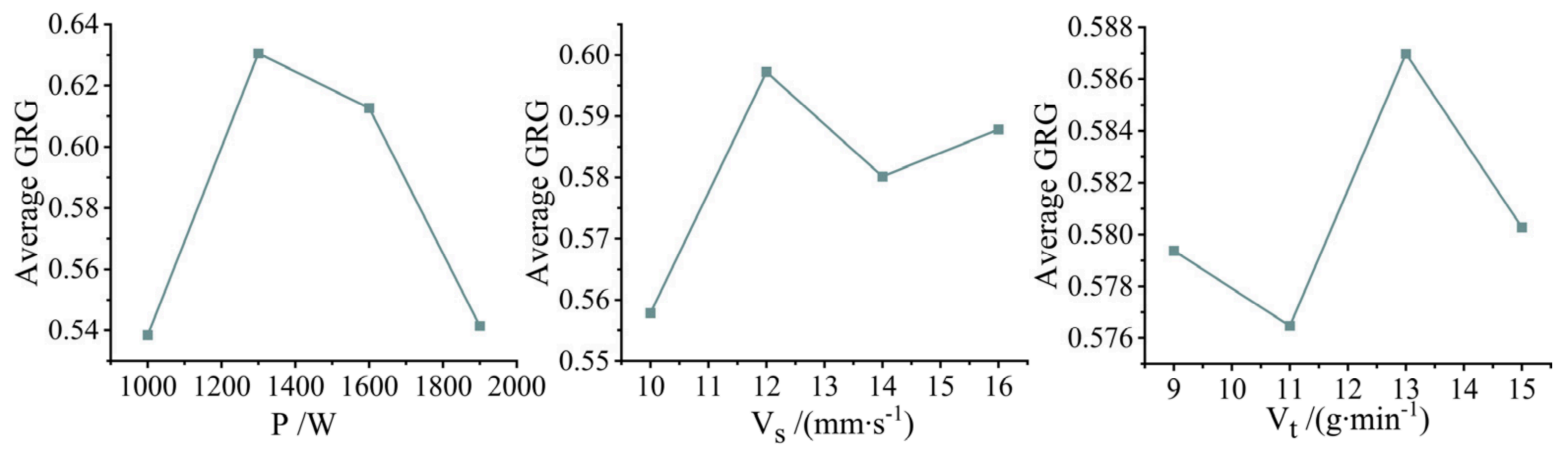
The main effects of the processing parameters on the mean GRG.

Figure 7 shows the main effects of laser power, scanning speed, and powder feed rate on the mean GRG. The mean GRG exhibited the largest variation across the laser power levels, followed by scanning speed, while the variation associated with powder feed rate was relatively small. According to the peak values of the main-effect curves for each factor, the optimized parameter combination was determined to be a laser power of 1300 W, a scanning speed of 12 mm/s, and a powder feed rate of 13 g/min.

**Figure 8:**
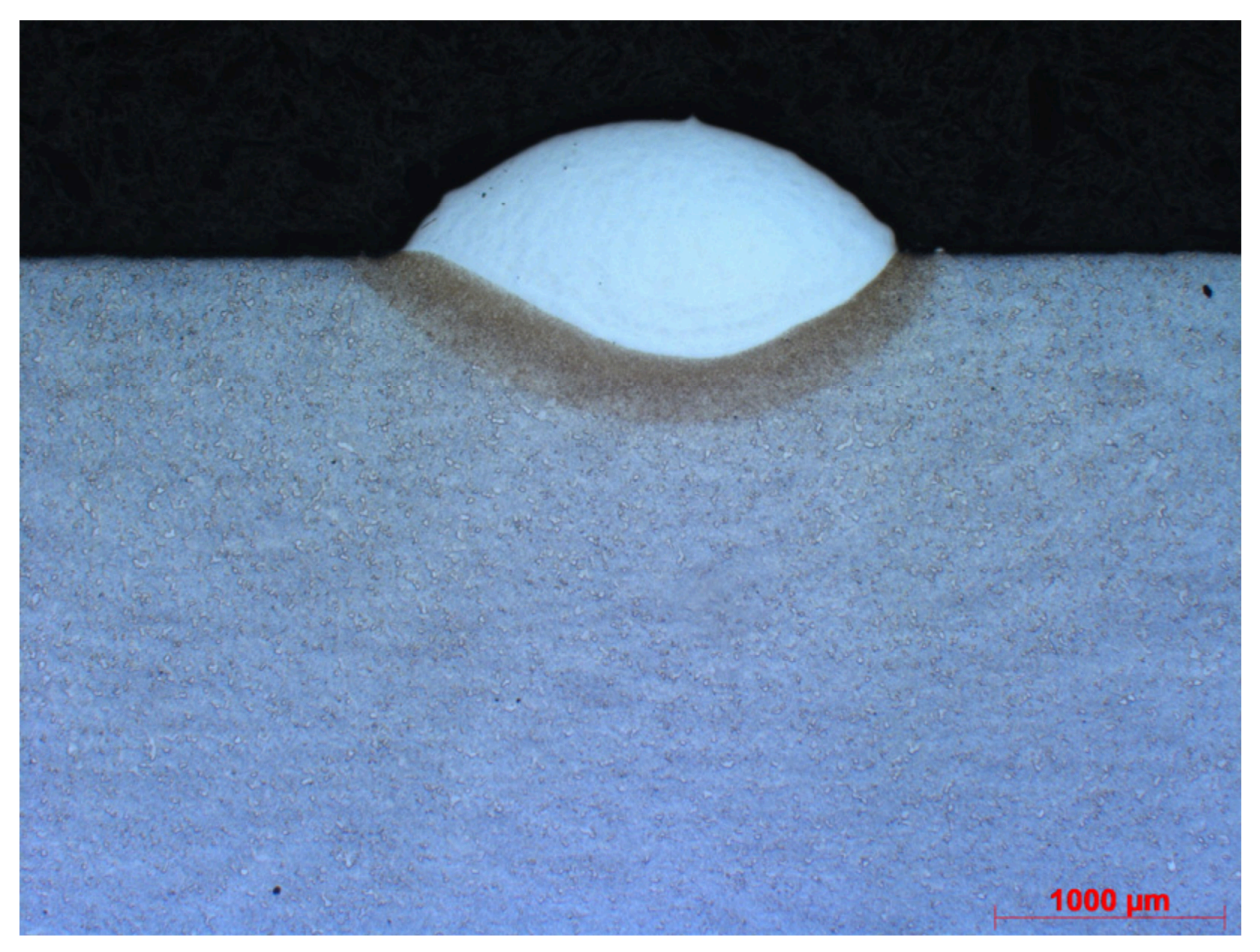
An optical micrograph of the clad layer cross-section from the confirmation experiment.

The above optimal parameter combination was determined based on the main-effect analysis of the average GRG values at different levels of each factor, rather than through regression models or other predictive models to calculate a theoretically predicted optimal GRG. Because the above parameter combination was not directly included in the L16 orthogonal experimental matrix, a confirmation experiment was further conducted. To ensure comparability of the evaluation results, the normalization criteria and entropy weights determined from the original orthogonal experiments were retained for comprehensive evaluation. As shown in Figure 8, in the confirmation experiment, the clad height, clad width, microhardness, surface roughness, and dilution ratio were 581.1 μm, 2353.5 μm, 910.4 HV, 4.832 μm, and 42%, respectively, corresponding to a GRG of 0.71087. This value was higher than the maximum GRG obtained in the orthogonal experiments, i.e., 0.67598 for specimen No. 9. Therefore, within the investigated parameter range of this study, 1300 W, 12 mm/s, and 13 g/min were selected as the base cladding parameters for subsequent remelting experiments and multi-track multilayer specimen fabrication.

### 3.2. Single-Track Remelting Results and Parameter Optimization

#### 3.2.1. Single-Track Remelting Results

**Figure 9:**
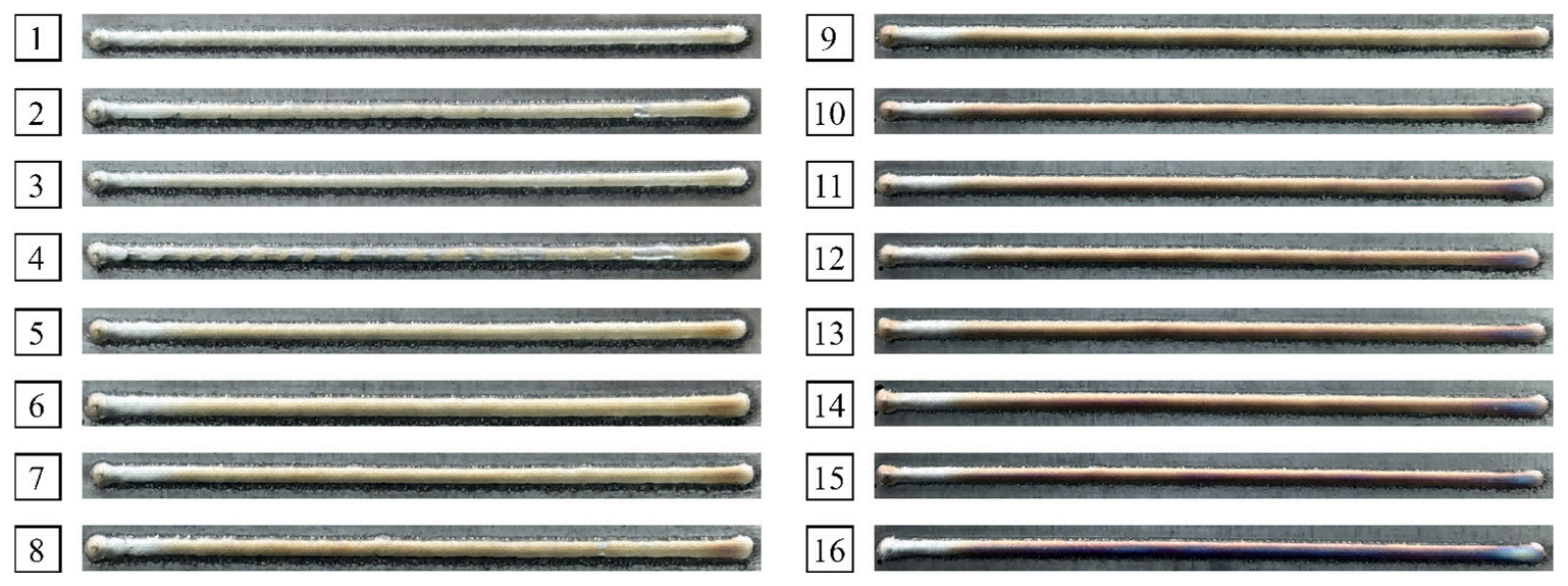
Macroscopic surface morphologies of single-track remelted clad layers.

**Figure 10:**
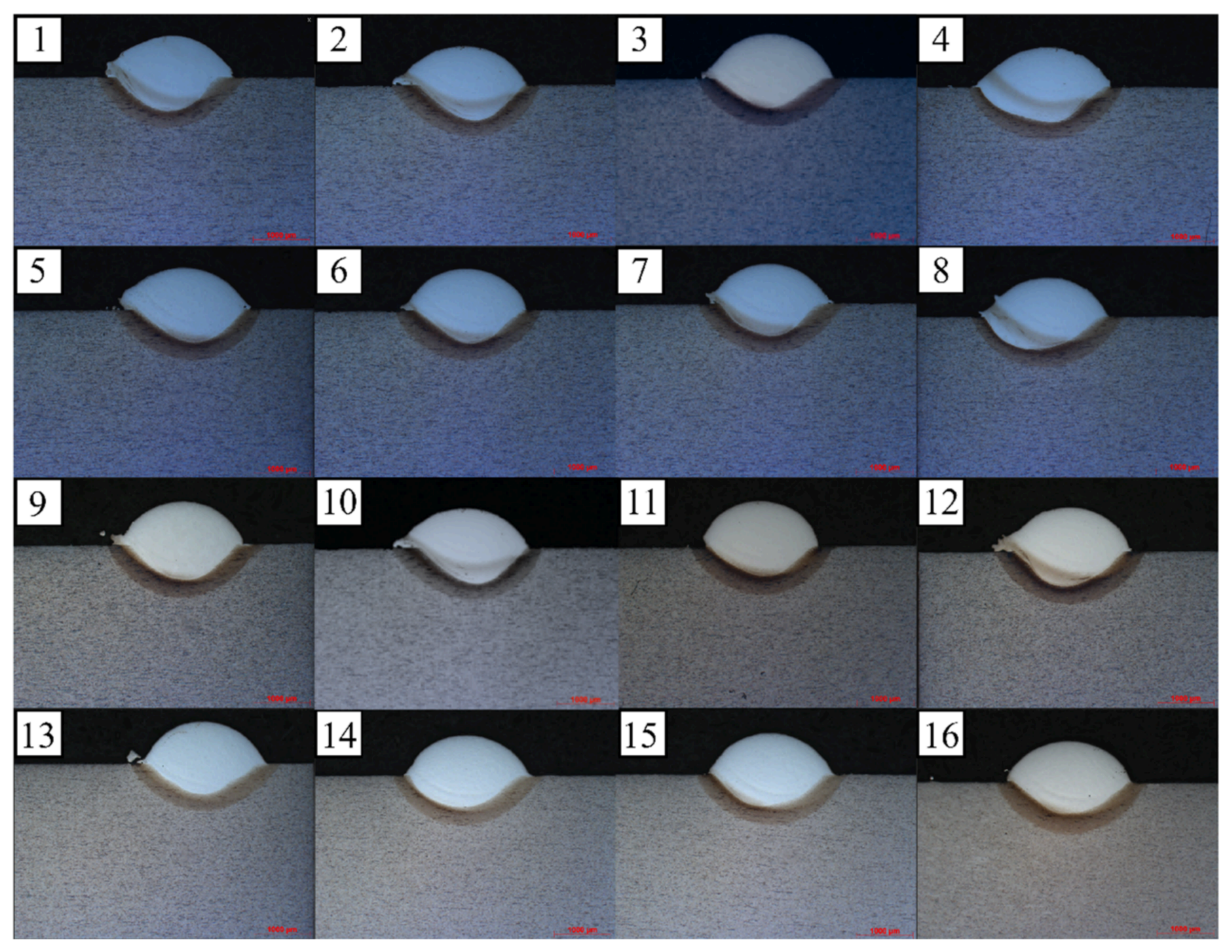
Optical micrographs of cross-sections of single-track remelted clad layers.

Figure 9 and Figure 10 show the surface and cross-sectional morphologies of the specimens under different remelting parameters, respectively. After remelting, adhered powder particles and local surface undulations on the clad tracks were noticeably reduced, and the track profiles became more continuous. This indicates that the redistribution and spreading of molten metal during remelting contribute to improved surface quality.

Table 5 presents the measured clad height H, clad width W, microhardness HV, surface roughness Sa, and dilution ratio η under different remelting parameter combinations (see Table S2 for full experimental data). The response indicators varied with the remelting parameters to different extents, indicating that the remelting laser power and scanning speed jointly affect the forming characteristics and properties of the clad layers.

#### 3.2.2. Comprehensive Evaluation and Optimization of Single-Track Remelting Parameters

**Figure 11:**
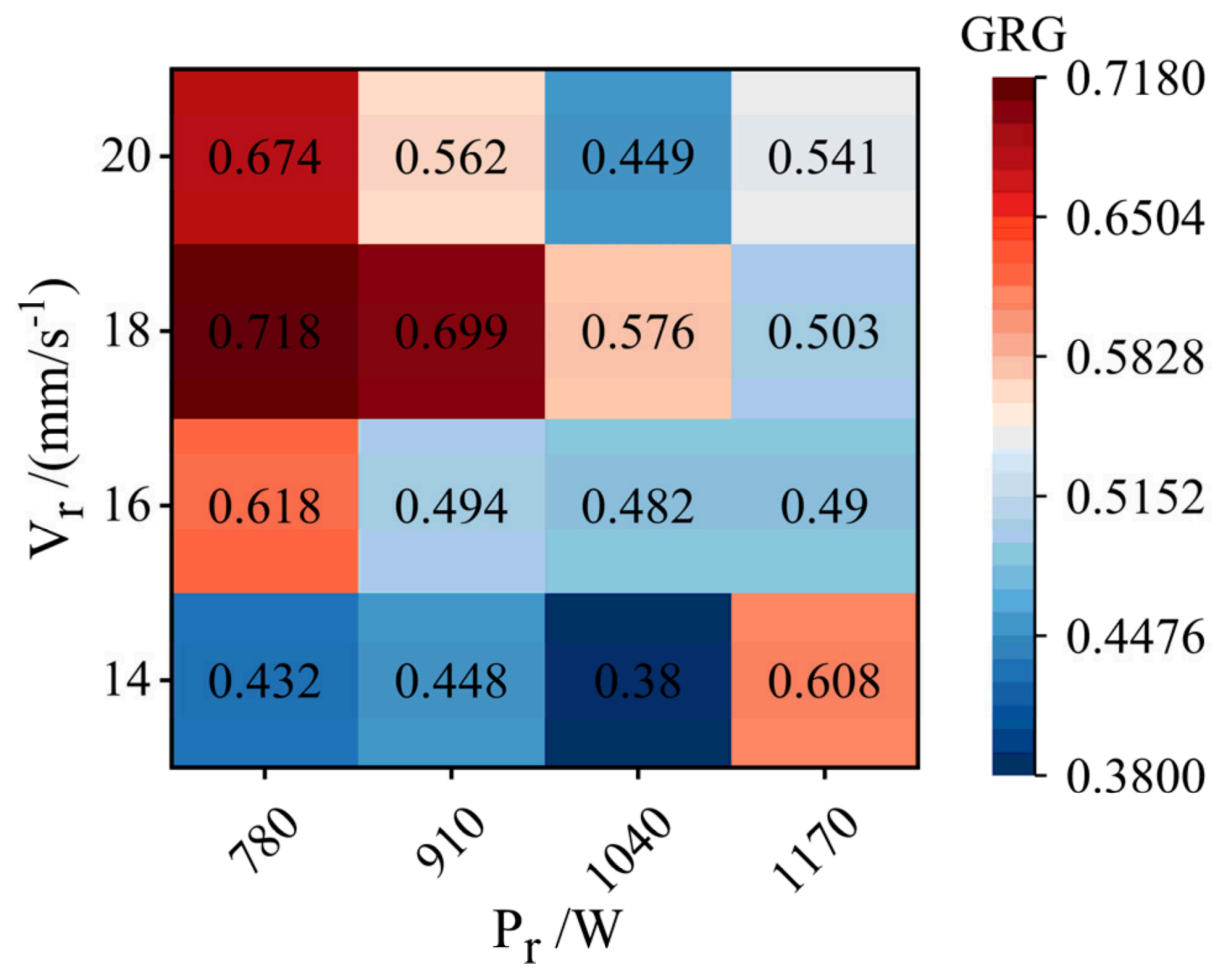
The distribution of the gray relational grade (GRG) under different remelting parameter combinations.

To determine the preferred parameter combination for single-track remelting within the investigated parameter range, the EWM-GRA method described above was applied to comprehensively evaluate the multiple response indicators listed in Table 5. The resulting GRG distribution is shown in Figure 11. Considerable differences in GRG were observed among the different parameter combinations. Specimen No. 3 exhibited the highest GRG of 0.71816, corresponding to a remelting laser power of 780 W and a scanning speed of 18 mm/s. This parameter combination provided a relatively favorable balance among geometrical characteristics, dilution ratio, microhardness, and surface roughness within the investigated remelting parameter range.

**Figure 12:**
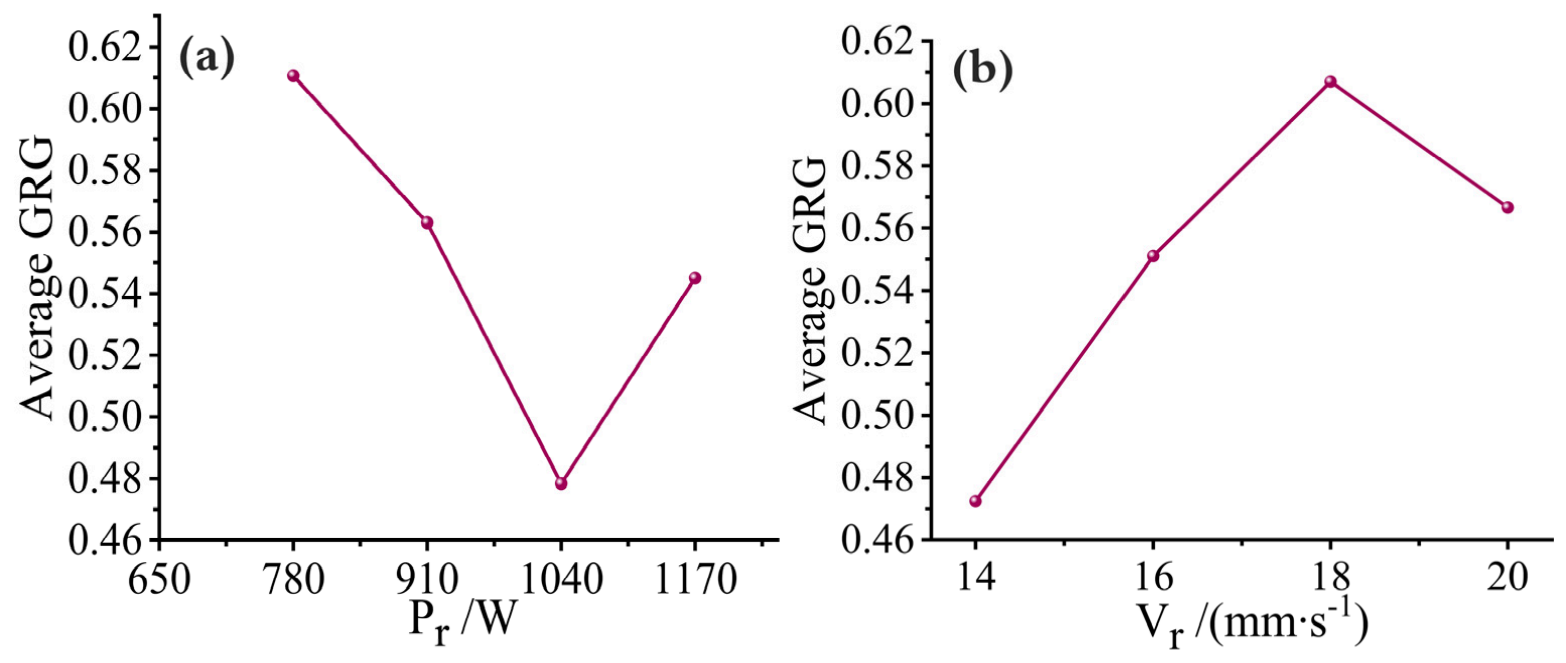
The main effects of the remelting parameters on the mean GRG: (**a**) remelting laser power; (**b**) remelting scanning speed.

A main-effect analysis was further conducted using the GRG as the response, as shown in Figure 12. The mean GRG varied with both remelting laser power and scanning speed, with a slightly greater variation observed for scanning speed. Based on the peak values of the main-effect curves, the optimal remelting parameters were determined to be a laser power of 780 W and a scanning speed of 18 mm/s. Since this parameter combination was already included in the 16-run full-factorial design and its experimental response was directly available, no additional confirmation experiment was required. These parameters were therefore adopted for the subsequent layer-by-layer remelting of the multi-track multilayer specimens.

**Figure 13:**
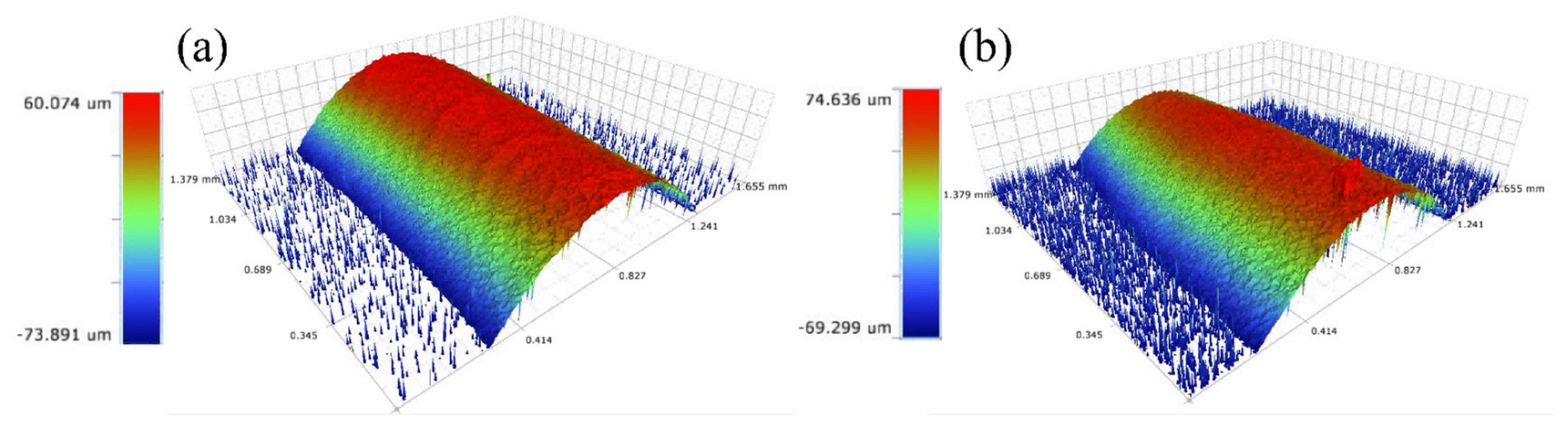
Three-dimensional surface topographies of single-track clad layers obtained by white light interferometry: (**a**) remelted specimen; (**b**) non-remelted specimen.

To further evaluate the surface improvement achieved under the optimized remelting parameters, the three-dimensional surface topographies of remelted specimen No. 3 and the non-remelted specimen were characterized, as shown in Figure 13. Compared with the non-remelted specimen, the remelted specimen exhibited reduced surface height fluctuations and less pronounced local protrusions, resulting in a smoother overall surface profile. Meanwhile, the areal surface roughness Sa decreased from 4.832 μm to 1.953 μm. These results indicate that under the optimized parameters, the redistribution and spreading of molten metal during remelting contributed to improved surface flatness.

### 3.3. Comparison of the Forming Characteristics of Multi-Track Multilayer Clad Layers

**Figure 14:**
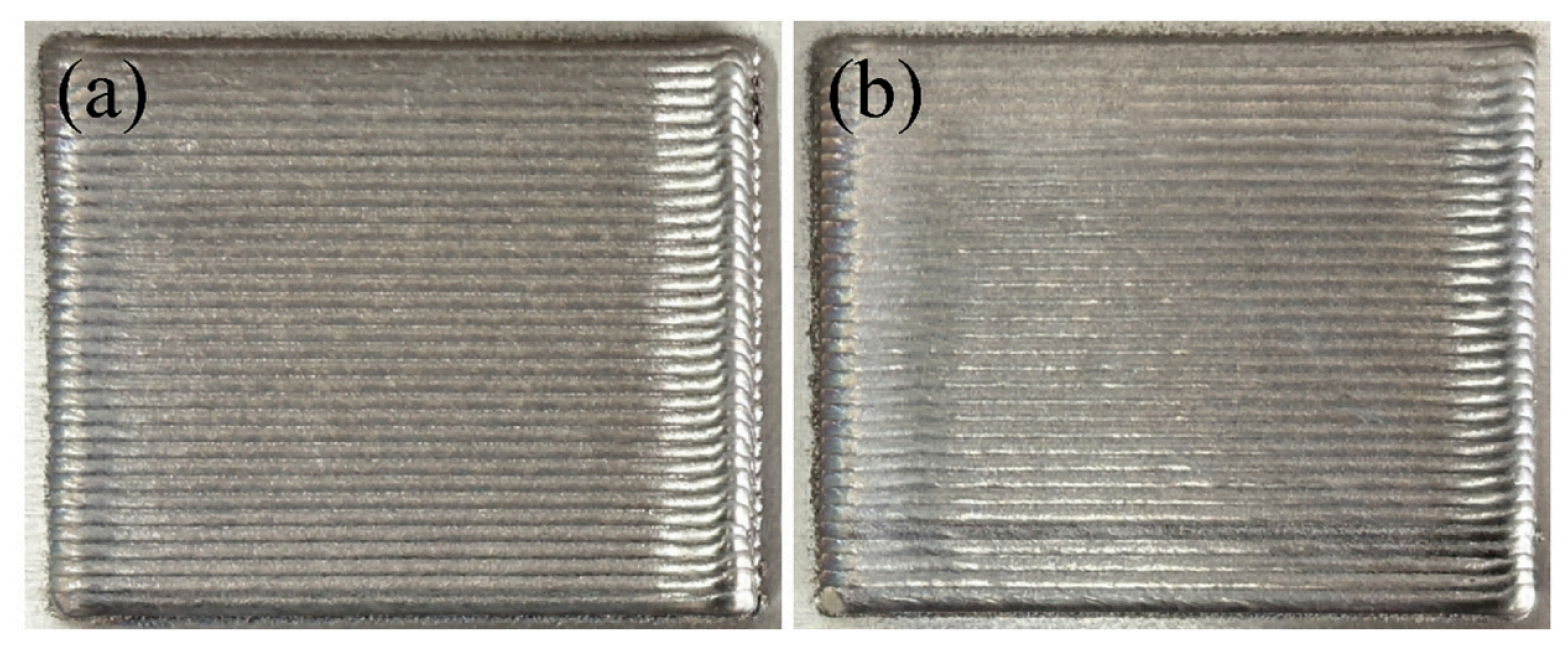
Macroscopic surface morphologies of multi-track multilayer specimens: (**a**) non-remelted specimen; (**b**) layer-by-layer remelted specimen.

Figure 14 compares the macroscopic surface morphologies of the multi-track multilayer specimens fabricated with and without layer-by-layer remelting. Both specimens exhibited continuous and complete multi-track multilayer structures. Compared with the non-remelted specimen, the layer-by-layer remelted specimen showed a flatter surface, with noticeably reduced track ripples and inter-track overlap traces, indicating that layer-by-layer remelting effectively improved the surface quality of the multi-track multilayer clad layer.

### 3.4. Microstructure and Mechanical Properties of Multi-Track Multilayer Specimens

#### 3.4.1. Microstructural Analysis

**Figure 15:**
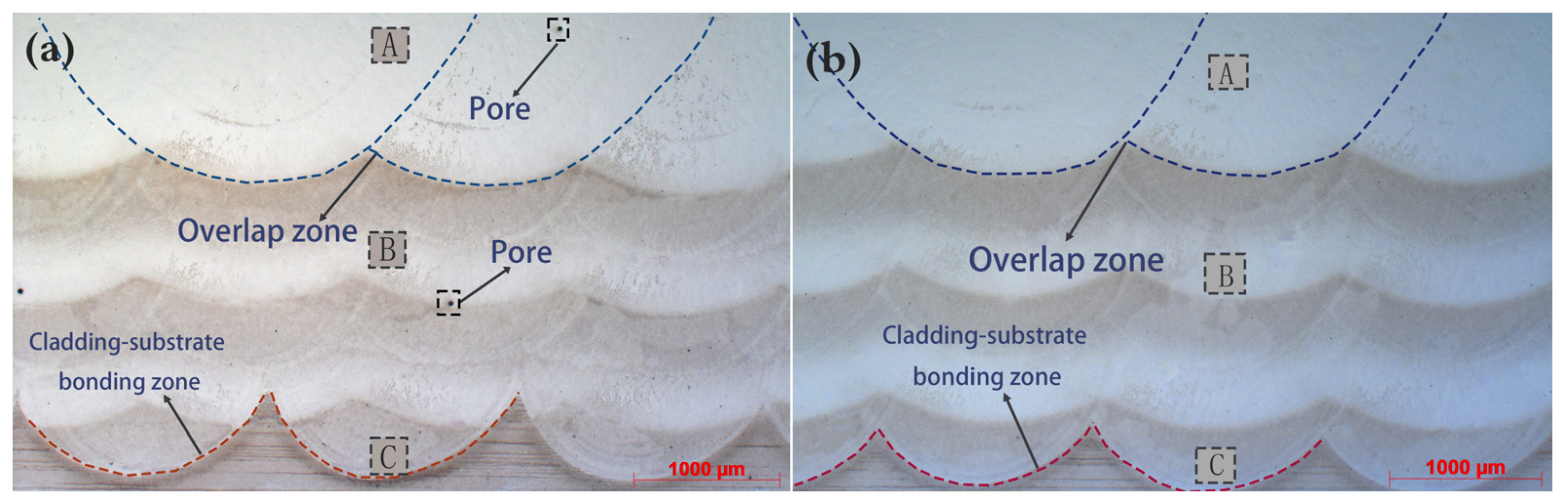
Overall cross-sectional morphologies and observation regions of multi-track multilayer clad layers: (**a**) non-remelted specimen; (**b**) layer-by-layer remelted specimen.

Figure 15 shows the overall cross-sectional morphologies of the multi-track multilayer specimens with and without layer-by-layer remelting. In the non-remelted specimen, the molten pool boundaries between adjacent layers were relatively distinct, and a small number of local pores were observed. By comparison, the layer-by-layer remelted specimen exhibited less pronounced molten pool boundaries, and qualitative observations of the selected cross-section indicated a tendency toward fewer locally observed pores. To further compare the microstructural characteristics at different heights along the deposition direction, the top region (A), middle region (B), and bottom region (C) were selected for higher-magnification observation.

**Figure 16:**
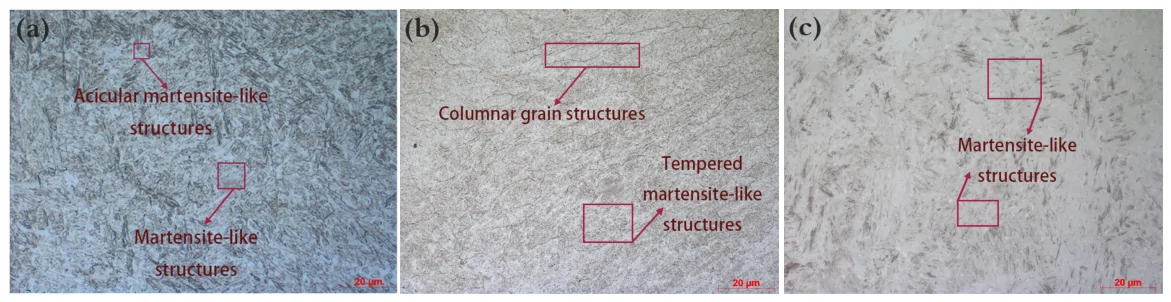
Microstructures at different regions of non-remelted multi-track multilayer clad layer: (**a**) top region; (**b**) middle region; (**c**) bottom region.

Figure 16 shows the high-magnification microstructures at different locations along the deposition direction of the non-remelted specimen. The top region exhibited an acicular martensite-like microstructure, reflecting the characteristics of rapid solidification. In the middle region, distinct columnar grain morphology was observed together with a tempered martensite-like microstructure. The bottom region, adjacent to the H13 steel substrate, exhibited a relatively fine martensite-like microstructure. Overall, pronounced microstructural variations were observed along the deposition direction in the non-remelted specimen, which may be attributed to differences in thermal history and heat dissipation conditions experienced by regions at different heights during multi-track multilayer deposition.

**Figure 17:**
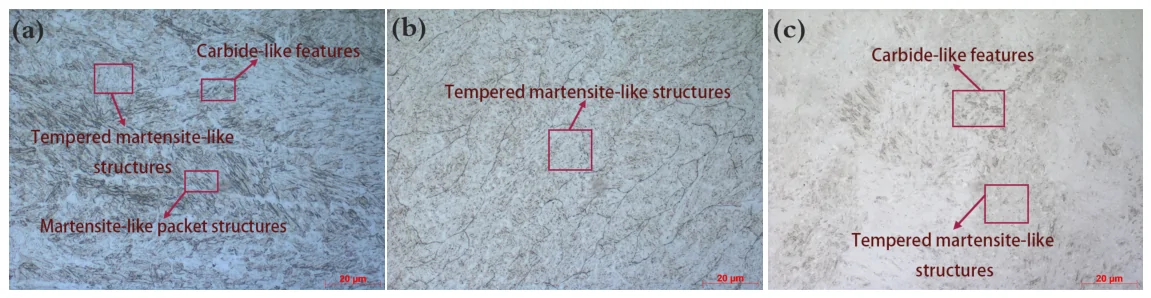
Microstructures in different regions of layer-by-layer remelted multi-track multilayer clad layer: (**a**) top region; (**b**) middle region; (**c**) bottom region.

After layer-by-layer remelting, the microstructures in the different regions changed, as shown in Figure 17. The top region was mainly characterized by a tempered martensite-like microstructure accompanied by fine carbide-like features, while the middle region exhibited a relatively uniform tempered martensite-like microstructure. Similar tempered martensite-like microstructures and fine carbide-like features were also observed in the bottom region. Compared with the non-remelted specimen, the directionality of columnar grains in the middle region was noticeably reduced after layer-by-layer remelting, and the differences in microstructural morphology among regions at different heights were decreased. These observations may be attributed to the remelting–resolidification process and repeated thermal cycling induced by layer-by-layer remelting, which may promote microstructural modification and reduce the differences in microstructural morphology among regions at different heights.

#### 3.4.2. Microhardness Analysis

**Figure 18:**
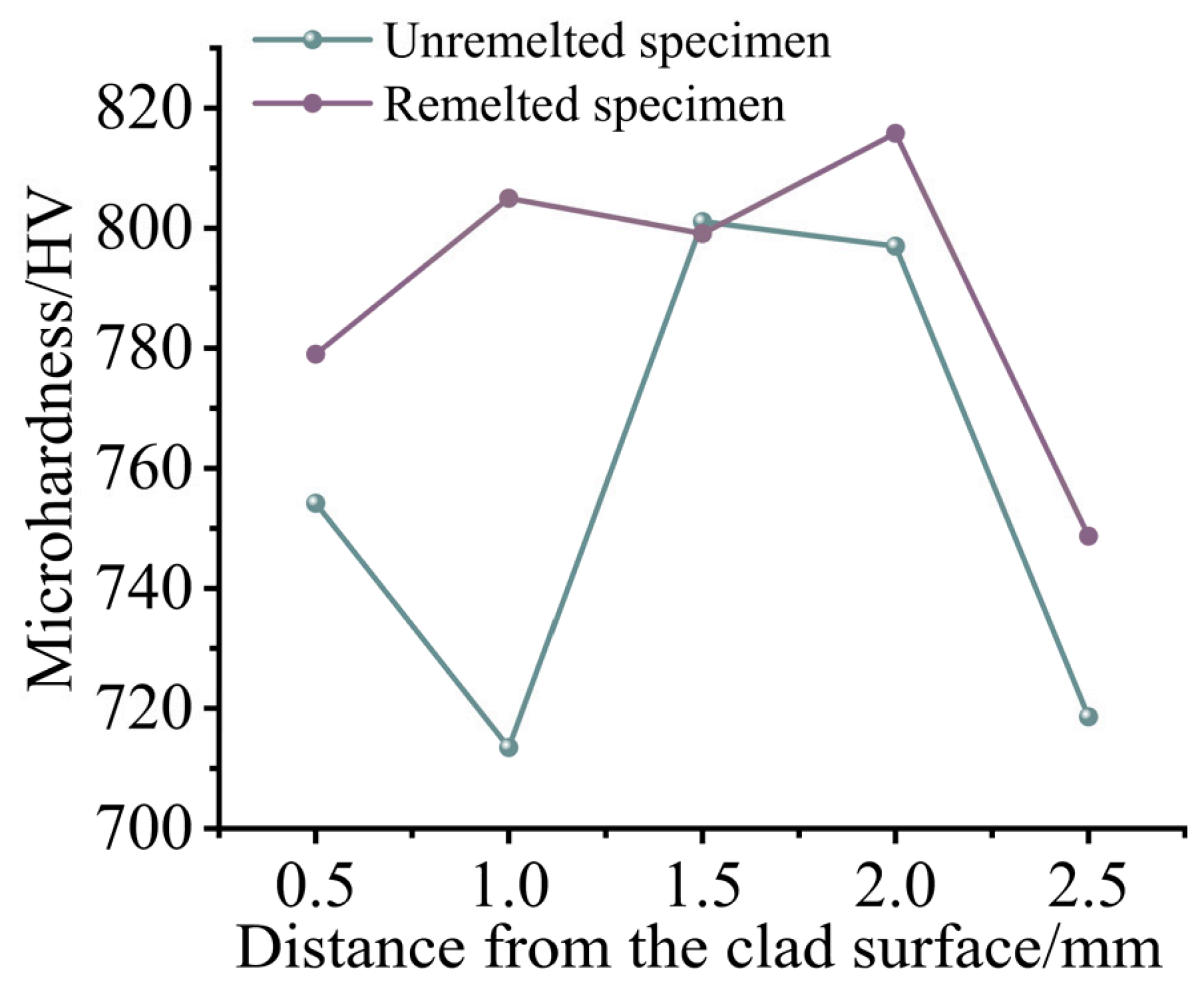
Microhardness distributions along the thickness direction of the multi-track multilayer specimens.

Figure 18 shows the microhardness distributions along the thickness direction of the clad layers for the non-remelted and layer-by-layer remelted specimens. The hardness values of both specimens fluctuated to some extent with measurement position; however, the layer-by-layer remelted specimen exhibited a higher average microhardness at the measured positions. The average microhardness values calculated from the five measurement points were 756.9 HV for the non-remelted specimen and 789.5 HV for the layer-by-layer remelted specimen. It should be noted that these average values were calculated from five fixed measurement points along the thickness direction of a single specimen under each processing condition. They were used only to represent the overall hardness level of the measured region rather than independent specimen replicates; therefore, no statistical significance comparison was performed. In particular, the layer-by-layer remelted specimen reached the highest microhardness value within the measured region (815.8 HV) at a position 2.0 mm below the top surface of the clad layer.

The increase in average microhardness after layer-by-layer remelting may be associated with microstructural modification induced by remelting–resolidification and repeated thermal cycling. As indicated by the preceding microstructural observations, layer-by-layer remelting reduced the differences in microstructural morphology among regions at different heights, and fine carbide-like features were also observed. These microstructural changes may have contributed to the increase in the overall hardness of the clad layer. Overall, the layer-by-layer remelting specimens exhibited a higher average microhardness at the measured positions, although a certain degree of hardness fluctuation remained along the thickness direction.

#### 3.4.3. Tensile Properties

Table 6 summarizes the room-temperature tensile properties of the two groups of specimens. The layer-by-layer remelted specimens exhibited higher strength levels, with the average ultimate tensile strength increasing from 1524.23 MPa to 1604.46 MPa, corresponding to an increase of approximately 5.3%, and the average yield strength increasing from 1173.37 MPa to 1342.88 MPa, corresponding to an increase of approximately 14.4%. Meanwhile, the average elongation after fracture decreased from 2.57% to 1.41%. These changes in mechanical properties may be associated with the observed tendency toward reduced local pores, reduced differences in microstructural morphology among regions at different heights, and microstructural changes induced by repeated thermal cycling during layer-by-layer remelting.

During layer-by-layer remelting, the clad layer undergoes remelting and resolidification. The observed tendency toward reduced local pores may help alleviate defect-induced local stress concentrations and may contribute to the improvement in strength. Meanwhile, the reduced differences in microstructural morphology among regions at different heights after layer-by-layer remelting may also be related to the changes in mechanical properties. However, the decrease in elongation after fracture of the layer-by-layer remelted specimens indicates a reduction in ductility, which may be related to the increased degree of local hardening and fracture morphology changes. In addition, the repeated heating and cooling cycles induced by layer-by-layer remelting may alter the residual stress state within the clad layer and potentially influence its strength and ductility. Since residual stress was not measured in this study, its specific contribution remains unclear.

#### 3.4.4. Fracture Morphology Analysis

**Figure 19:**
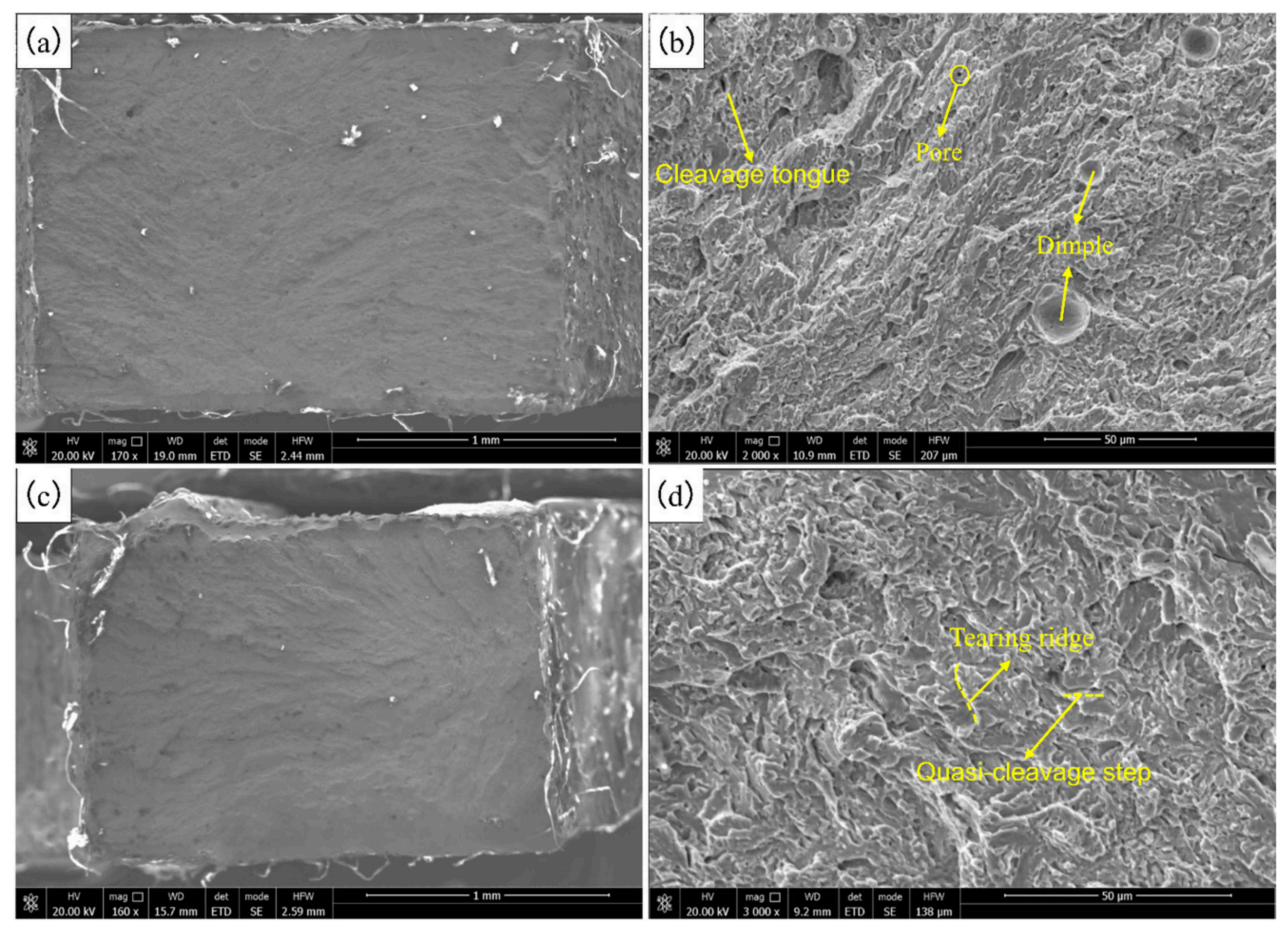
Fracture morphologies of specimens: (**a**) non-remelted specimen at low magnification; (**b**) non-remelted specimen at high magnification; (**c**) layer-by-layer remelted specimen at low magnification; (**d**) layer-by-layer remelted specimen at high magnification.

Figure 19 shows the tensile fracture morphologies of the non-remelted and layer-by-layer remelted specimens. Figure 19a,b correspond to the low- and high-magnification fracture surfaces of the non-remelted specimen, respectively, while Figure 19c,d show the corresponding morphologies of the layer-by-layer remelted specimen. At low magnification, both specimens exhibited relatively flat fracture surfaces without obvious necking, indicating limited overall plastic deformation of the two clad structures.

At higher magnification, the fracture surface of the layer-by-layer remelted specimen exhibited numerous quasi-cleavage steps and tearing ridges, while dimples were relatively less evident, indicating a mixed fracture mode dominated by quasi-cleavage features. In contrast, pores, cleavage tongues, and localized dimples were observed on the fracture surface of the non-remelted specimen, with the dimples indicating a certain degree of local plastic deformation during fracture. These fracture characteristics are consistent with the tensile results. The higher strength of the layer-by-layer remelted specimen corresponds to the previously observed tendency toward reduced local pores and improved microstructural uniformity, whereas the more pronounced quasi-cleavage features are consistent with its reduced elongation after fracture.

It should be noted that this study still has certain limitations. The variations in pores and microstructural differences were mainly evaluated based on qualitative observations, and further quantitative characterization is still required. In addition, the effects of residual stress and interactions among processing parameters were not systematically investigated in this study. The optimized processing parameters obtained are only applicable within the investigated range of this work. Future studies will further verify these aspects.

## 4. Conclusions

This study investigated the effects of layer-by-layer remelting on the forming characteristics, microstructure, and mechanical properties of multi-track multilayer H13 steel clad structures using a combined laser cladding–layer-by-layer remelting process. The experimental procedure followed the sequence of basic cladding parameter optimization–remelting parameter optimization–multi-track multilayer specimen evaluation. The main conclusions are as follows:

(1) The basic cladding parameters were comprehensively evaluated using an L16 (4^3^) orthogonal design combined with the EWM-GRA method. Within the investigated parameter range, the mean GRG exhibited the largest variation across the laser power levels, followed by scanning speed, whereas the variation associated with powder feed rate was relatively small. The optimal basic cladding parameters were determined to be a laser power of 1300 W, a scanning speed of 12 mm/s, and a powder feed rate of 13 g/min. The confirmation experiment yielded a GRG of 0.71087, which was higher than the maximum value of 0.67598 obtained among the 16 orthogonal experiments.
(2) Under the optimized basic cladding parameters, a two-factor, four-level full-factorial experiment was conducted to optimize the remelting laser power and scanning speed. The EWM-GRA results showed that specimen No. 3 achieved the highest GRG of 0.71816. Within the investigated remelting parameter range, the optimized remelting parameters were determined to be a laser power of 780 W and a scanning speed of 18 mm/s. After remelting under these conditions, the surface roughness Sa of the single-track clad layer decreased from 4.832 μm to 1.953 μm, accompanied by reduced surface height fluctuations and less pronounced local protrusions, indicating that remelting effectively improved the surface flatness of the clad track.
(3) After layer-by-layer remelting, the multi-track multilayer specimens exhibited a flatter surface with reduced track ripples and inter-track overlap traces. Cross-sectional qualitative observations indicated a tendency toward reduced local pores. Meanwhile, the directionality of columnar grains in the middle region was reduced, and the differences in microstructural morphology among regions at different heights were decreased.
(4) The layer-by-layer remelted specimen exhibited a higher average microhardness at the measured positions. The average microhardness values calculated from the five measurement points were 756.9 HV for the non-remelted specimen and 789.5 HV for the layer-by-layer remelted specimen. The average ultimate tensile strength increased from 1524.23 MPa to 1604.46 MPa, and the average yield strength increased from 1173.37 MPa to 1342.88 MPa. Meanwhile, the average elongation after fracture decreased from 2.57% to 1.41%. These results indicate that layer-by-layer remelting improved the strength of the specimens while reducing their ductility.

## Figures and Tables

**Table 1:** Nominal chemical composition range of H13 steel.

Element	C	Si	Mn	Cr	Mo	V	P	S
wt/%	0.35~0.45	0.80~1.20	0.20~0.50	4.75~5.50	1.10~1.75	0.80~1.20	≤0.03	≤0.03

**Table 2:** Orthogonal experimental design.

No.	*P*/W	*V_s_*/(mm∙s^−1^)	*V_t_*/(g∙min^−1^)
1	1000	10	9
2	1000	12	11
3	1000	14	13
4	1000	16	15
5	1300	10	11
6	1300	12	9
7	1300	14	15
8	1300	16	13
9	1600	10	13
10	1600	12	15
11	1600	14	9
12	1600	16	11
13	1900	10	15
14	1900	12	13
15	1900	14	11
16	1900	16	9

**Table 3:** Two-factor, four-level full-factorial experimental design.

No	*P_r_*/W	*V_r_*/(mm∙s^−1^)	No	*P_r_*/W	V_r_/(mm∙s^−1^)
1	780	14	9	1040	14
2	780	16	10	1040	16
3	780	18	11	1040	18
4	780	20	12	1040	20
5	910	14	13	1170	14
6	910	16	14	1170	16
7	910	18	15	1170	18
8	910	20	16	1170	20

**Table 4:** Measured response values of single-track cladding orthogonal experiments.

No	H (μm)	W (μm)	HV	Sa (μm)	η (%)
1	509.3	2112.1	872.17	9.182	48.8
2	494.1	2018.2	942.83	9.437	47.8
3	490.5	2044.1	918.33	8.555	44.3
4	557.3	1903.8	909.30	10.06	33.1
5	709.7	2370.8	870.77	5.185	44.3
6	511.3	2369.4	878.47	4.837	53.8
7	667.1	2257.3	935.23	6.002	42.9
8	523.2	2132.5	848.63	5.193	42.7
9	841.3	2703.8	860.5	4.817	41.6
10	809.8	2614.5	854.23	5.181	41.3
11	451.5	2602.0	828.17	5.772	62.3
12	502.5	2390.8	824.03	5.532	49.8
13	1039.3	2790.1	865.97	9.604	45.5
14	811.6	2764.3	888.4	8.532	50.5
15	597.9	2944.6	837.8	8.946	57.9
16	478.0	2858.6	924.17	9.456	58.5

**Table 5:** Measured response values under different remelting parameter combinations.

No	H (μm)	W (μm)	HV	Sa (μm)	η (%)
1	715.0	2215.1	840.5	3.015	45.5
2	656.9	2181.8	912.3	2.264	44.6
3	671.7	2232.8	993.8	1.953	43.3
4	688.9	2302.1	1029.5	4.448	45.2
5	706.8	2247.6	856.9	3.439	45.1
6	711.4	2125.8	976.7	3.422	44.9
7	661.0	2182.5	1033.2	3.942	46.3
8	674.5	2256.3	966.6	3.142	45.4
9	741.5	2253.9	851.4	4.078	45.1
10	745.7	2290.9	891.3	3.963	40.8
11	760.8	2019.6	854.1	4.576	40.6
12	759.9	2239.7	927.8	5.022	43.9
13	734.1	2139.9	983.5	3.408	41.2
14	690.1	2245.2	885.4	3.324	43.7
15	686.9	2200.5	861.3	3.246	43.0
16	697.5	2171.4	851.3	2.209	41.6

**Table 6:** Room-temperature tensile properties of the multi-track multilayer specimens.

Specimen	Ultimate Tensile Strength (MPa)	Yield Strength (MPa)	Elongation after Fracture (%)
Non-remelted	1524.23 ± 57.16	1173.37 ± 49.72	2.57 ± 0.52
Layer-by-Layer Remelted	1604.46 ± 39.31	1342.88 ± 45.26	1.41 ± 0.27

## Data Availability

The data supporting the findings of this study are included in the article/Supplementary Materials. Further inquiries can be directed to the corresponding author.

## Supplementary Materials

The following supporting information can be downloaded at: https://www.mdpi.com/article/10.3390/ma19194196/s1, Table S1: Measured response values of the single-track cladding orthogonal experiments. Table S2: Measured response values under different remelting parameter combinations.
