# Supplementary material for: The Effects of Combined Laser Cladding and Layer-by-Layer Remelting on the Forming Quality and Properties of H13 Steel Clad Layers
Source: Materials (Basel). 2026 Sep 30;19(19):4196. doi: 10.3390/ma19194196 (PMC13665957; doi:10.3390/ma19194196)
Supplement: Supplementary file 1 [file materials-19-04196-s001.zip › materials-4589997-supplementary.pdf]

Supplementary Data for EWM-GRA Calculation

The original unrounded experimental data used for the EWM-GRA calculation are provided in this supplementary file. The data include the measured responses of clad height (H), clad width (W), microhardness (HV), surface roughness (Sa), and dilution ratio (η) obtained from the 16 experimental groups.

To avoid the influence of numerical rounding on the optimization results, all entropy weights, gray relational coefficients, and gray relational grades (GRGs) were calculated using the original unrounded data. The values reported in Tables 4 and 5 of the revised manuscript were rounded only for presentation purposes.

Table S1. Measured response values of the single-track cladding orthogonal experiments.

No	H (μm)	W (μm)	HV	Sa (μm)	η (%)
1	509.346	2112.117	872.17	9.182	48.836
2	494.095	2018.193	942.83	9.437	47.799
3	490.508	2044.079	918.33	8.555	44.321
4	557.269	1903.775	909.30	10.06	33.121
5	709.713	2370.779	870.77	5.185	44.298
6	511.303	2369.408	878.47	4.837	53.849
7	667.143	2257.279	935.23	6.002	42.927
8	523.174	2132.539	848.63	5.193	42.757
9	841.338	2703.810	860.5	4.817	41.597
10	809.974	2614.496	854.23	5.181	41.295
11	451.531	2602.041	828.17	5.772	62.335
12	502.465	2390.841	824.03	5.532	49.842
13	1039.267	2790.143	865.97	9.604	45.543
14	811.583	2764.359	888.4	8.532	50.497
15	597.946	2944.649	837.8	8.946	57.911
16	478.014	2858.629	924.17	9.456	58.475

Table S2. Measured response values under different remelting parameter combinations.

No	H (μm)	W (μm)	HV	Sa (μm)	η (%)
1	715.043	2215.123	840.5	3.015	45.492
2	656.896	2181.804	912.3	2.264	44.559
3	671.652	2232.796	993.8	1.953	43.319
4	688.946	2302.088	1029.5	4.448	45.214
5	706.822	2247.634	856.9	3.439	45.057
6	711.434	2125.839	976.7	3.422	44.979
7	661.006	2182.474	1033.2	3.942	46.262
8	674.545	2256.311	966.6	3.142	45.413
9	741.462	2253.865	851.4	4.078	45.112
10	745.665	2290.918	891.3	3.963	40.771
11	760.769	2019.552	854.1	4.576	40.598
12	759.942	2239.739	927.8	5.022	43.912
13	734.076	2139.968	983.5	3.408	41.251
14	690.080	2245.280	885.4	3.324	43.723
15	686.901	2200.494	861.3	3.246	43.009
16	697.552	2171.367	851.3	2.209	41.653
